# Development of a dynamic framework to explain population patterns of leisure-time physical activity through agent-based modeling

**DOI:** 10.1186/s12966-017-0553-4

**Published:** 2017-08-22

**Authors:** Leandro M. T. Garcia, Ana V. Diez Roux, André C. R. Martins, Yong Yang, Alex A. Florindo

**Affiliations:** 1 University of Sao Paulo School of Public Health, Sao Paulo, Brazil; 2 Drexel University Dornsife School of Public Health, Philadelphia, USA; 3 University of Sao Paulo School of Arts, Sciences and Humanities, Sao Paulo, Brazil; 4 University of Memphis School of Public Health, Memphis, USA

**Keywords:** Physical activity, Theoretical models, Framework, Systems science, Social-ecological models, Agent-based model

## Abstract

Despite the increasing body of evidences on the factors influencing leisure-time physical activity, our understanding of the mechanisms and interactions that lead to the formation and evolution of population patterns is still limited. Moreover, most frameworks in this field fail to capture dynamic processes. Our aim was to create a dynamic conceptual model depicting the interaction between key psychological attributes of individuals and main aspects of the built and social environments in which they live. This conceptual model will inform and support the development of an agent-based model aimed to explore how population patterns of LTPA in adults may emerge from the dynamic interplay between psychological traits and built and social environments. We integrated existing theories and models as well as available empirical data (both from literature reviews), and expert opinions (based on a systematic expert assessment of an intermediary version of the model). The model explicitly presents intention as the proximal determinant of leisure-time physical activity, a relationship dynamically moderated by the built environment (access, quality, and available activities) – with the strength of the moderation varying as a function of the person’s intention– and influenced both by the social environment (proximal network’s and community’s behavior) and the person’s behavior. Our conceptual model is well supported by evidence and experts’ opinions and will inform the design of our agent-based model, as well as data collection and analysis of future investigations on population patterns of leisure-time physical activity among adults.

**Electronic supplementary material:**

The online version of this article (doi:10.1186/s12966-017-0553-4) contains supplementary material, which is available to authorized users.

## Introduction

In consonance with the increasing body of evidence regarding the impact of environmental factors, theories and conceptual models on the adoption and maintenance of physical activity practice have been transitioning from focusing on psychological factors to social-ecological approaches, which posit the existence of several levels of influence on the behavior. These levels of influence include intrapersonal, interpersonal, physical environment, and policy factors, among others [1, 2]. Social-ecological approaches also recognize individuals as part of larger social systems, and interactions between individuals and environments as essential drivers of health events [2].

Despite the substantial progress made on expanding our understanding on the multiple factors, at multiple levels, influencing physical activity, current theories and models still lack some features that may be critical to understanding the formation and evolution of population patterns. First, most of the theories and models are acyclic, that is, they fail to capture dynamic processes by which the behavior, intrapersonal attributes, built and social environments are continuously and endogenously shaped. Physical activity is not a one-time behavior and the interdependent, adapting nature of the elements and processes involved in adopting and maintaining the behavior needs to be take into account [3]. Second, most social-ecological approaches demarcate boundaries between different levels and present elements within each level, but interactions and causal pathways between elements, either within or between levels, are rarely included [4].

To help to overcome these limitations, our purpose is to develop an agent-based model (ABM) to assist researchers to understand how the formation and evolution of leisure-time physical activity (LTPA) population patterns among adults occur. In this type of modeling, a system is represented as a composition of autonomous entities, called agents, the environment in which they live, and interactions between the parts. Each agent possesses decision-making capabilities, determined by a set of rules on how to act, derived from his perceptions of the surrounding environment and the interaction with other agents [5, 6]. Therefore, ABMs are useful tools to represent social systems and the interactions between individuals and with the environment in which they live. Furthermore, relationships and causal pathways between elements of different levels can be represented in a dynamic manner, enabling researchers to understand and explore the behavior of complex systems, such as the collective patterns of LTPA in human societies, from a socio-ecological perspective.

LTPA is one of the pillars for most population-based initiatives promoting physical activity. It is a complex, multidimensional behavior [7, 8], influenced by a network of factors interacting dynamically, such as attributes of the built environment (mixed land use, street connectivity, access to recreation facilities, residential density, aesthetics, and transport infrastructure [1, 9, 10]), aspects of the social environment (socioeconomic status [11], norms, social networks, social support, and social capital [12, 13]), and psychological traits (self-efficacy, intention [14, 15], habit [16], motivation and satisfaction [17, 18]). Moreover, literature on LTPA is vast and rich to support the modeling process. However, despite these reasons, all ABMs dedicated to physical activity published until now focused on transport-related walking patterns [19].

Our effort will start focusing on the dynamic interactions between the individuals’ psychological traits and the built and social environments in which they live. To support the ABM development, we had created a conceptual model depicting the interactions between key elements of these three levels (psychological, built and social environments) that may lead to population patterns of LTPA in adults. Since our assumptions impact the development and results of the ABM, in this paper we offer a complete, transparent, and detailed account of the process we took to obtain the current conceptual model.

## Methods

**Fig. 1:**
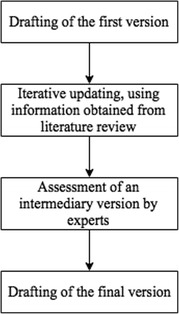
Conceptual model development process

Our ABM aims to capture the main psychological and environmental (built and social) elements and processes that may play a role in the LTPA pattern formation and evolution in adult populations. To support the ABM development, a conceptual model was built in four steps, depicted in Fig. 1. First, we drafted the first version (Additional file 1, Figure S1.1), based on the initial expertise of all authors. Second, we iteratively updated the model, using information obtained from literature review. Then, an intermediary version of the model (Additional file 2, Figure S2.1 and Table S2.1) was assessed by experts around the world. Finally, we drafted the resulting version, considering the expert assessments and new information found in the literature. The conceptual model development process lasted from November 2013 until May 2015.

The literature review aimed to consolidate, remove or include elements and relations within the conceptual model. We conducted searches in PubMed, Scopus, EBSCO and Web of Science utilizing terms related to LTPA constructs contained in the first version of the conceptual model (Additional file 1, Figure S1.1). We searched in titles, abstracts, and keywords using the following terms: “physical activity” OR exercise; theor* OR model*; behavi* AND (change OR adoption OR maintenance OR adherence); psycho* AND (characteristic OR attribute OR aspect OR correlate OR determinant); intention OR motivation OR attitude OR “perceived behav* control” OR “perceived competence” OR “self-efficacy” OR barrier; environment* AND (characteristic OR attribute OR aspect OR correlate OR determinant); “perceived environment” OR “built environment” OR “physical environment” OR “social environment” OR “social support” OR “subjective norm” OR “social influence” OR “social network”; socioeconomic OR income OR education* OR schooling OR deprivation OR poverty.

Manual searches were conducted for articles and books in the authors’ personal libraries as well. New sources of information were gradually added using a snowball process [20]. This new information was obtained from reading the selected sources, new searches on topics that were not initially included (e.g., perceived environment as mediator between built environment and LTPA), and identified through weekly alerts sent by the reference databases. Original empirical or theoretical works, meta-analysis, and systematic reviews published in peer-reviewed journals or books were selected.

In the development of the conceptual model, we sought theories and models that encompassed the interaction between psychological attributes, built and social environments, and human behavior, which helped us to delimit the elements and mechanisms and define constructs. Empirical evidence came mainly from meta-analyses and systematic reviews on elements and processes that might be retained, excluded or included in the conceptual model. The overall effect sizes reported by meta-analyses were interpreted as small (~0,2), average (~0,5), large (~0,8) or very large (~1,3 or higher) [21]. When effect sizes were not available, we considered the consistency and volume of the evidence reported, based on each review’s criteria. When meta-analysis or systematic reviews were absent, we searched for original empirical studies. Consistency of results between studies and effect size were considered in these cases. Whenever relevant, every effort was made to ensure the information referred to LTPA and to adult population (studies on institutionalized people have not been included). There was no geographical or date limitation for the searches. Only works published in English, Portuguese, or Spanish were reviewed. Search and extraction were conducted solely by the first author.

As for the expert-based assessment, our goal was to obtain inputs of around 20 experts of different fields. Expecting a response rate of 50%, we invited 40 researchers with recognized and recurrent international scientific production in topics relevant to the conceptual model (e.g., physical activity epidemiology or health-related built and social environments). We selected the experts among the authors of papers revised during the literature review process, as well as among authors of papers evaluated in the meta-analysis and systematic reviews we revised. Although all experts were PhDs, an effort was made to include junior and senior experts from around the world, to obtain more heterogeneous views. Experts’ descriptive information (sex, country, years as PhD, and fields of expertise) was obtained on their professional webpage or *curriculum vitae* by the first author.

The experts received individual e-mails inviting them for the assessment. Included in the emails were the intermediary version of the conceptual model (Additional file 2, Figure S2.1 and Table S2.1) and a link to an electronic assessment form. The conceptual model assessment consisted of three questions:

- How much do you agree or disagree with the current conceptual model?
- In your opinion, are there any variables or mechanisms not included in the current conceptual model that should be added?
- In your opinion, are there any variables or mechanisms included in the current model that should be excluded?

For the first question, experts informed their level of agreement through a five-point Likert scale (1 = completely disagree; 5 = completely agree). The two remaining questions were open ended. There was a space for additional comments as well. We asked the experts to assess and make suggestions based on the model’s purpose, goals and delimitations, to limit the answers’ content. We informed that the conceptual model would be used to support the development of an ABM with the purpose of exploring how the interaction between psychological traits and built and social environments leads to collective patterns of leisure-time physical activity practice in adults. Researchers had four weeks to send their contributions. We sent a reminder seven days before the deadline.

Initially, the first author read all the experts’ answers, to have a sense of their content. Secondly, he went back to each answer, extracting and grouping suggestions to include variables or mechanisms, exclude variables or mechanisms, or others. Then, suggestions were summarized based on content similarity. Suggestions were used to help decide whether elements and process should be kept, included or removed from the conceptual model, especially when referring to topics that had little or inconsistent evidence in literature. Recurring expert suggestions were prioritized. Only suggestions within the ABM’s current aims and scope (i.e., dynamic interplay between key psychological traits and built and social environments elements that may play a role in the LTPA population patterns) were considered for adoption in the following versions. Suggestions that contradicted good-quality and consistent evidence obtained through literature review or those outside the scope of the model (e.g., about demographic attributes) were not incorporated. Certain suggestions, although pertinent, were not adopted as we understood they would increase the model’s complexity beyond the desired level at the moment and were filed for future use as the ABM is further developed.

Experts’ descriptive information and results obtained on the Likert scale have been described in absolute frequencies. The content of the open-ended answers was read, analyzed and compiled by the first author.

The expert-based assessment was approved by the Ethics in Research Committee of the Sao Paulo University School of Arts, Sciences and Humanities (# 1.322.967).

## Results

The conceptual model includes elements of social practice theories [22, 23], the social-ecological model for physical activity [2], the integrated behavior change model for physical activity [24] and opinion dynamics models [25, 26]. Nineteen meta-analysis and systematic reviews were consulted during the development of the model. Additional file 3 reports full results from the literature review.

Of the 40 experts consulted, 18 (40%) sent their assessment. Among those, six were female. Six were working in Brazil, five in Australia, four in the United States, and three in Europe. Four were PhD for five years or less, none for six to nine years, five for 10 to 14 years, and nine for at least 15 years. The fields of expertise were diverse: physical activity epidemiology (*n* = 16), public health (*n* = 13), evaluation and translation of evidence-based health interventions (*n* = 10), health-related built and social environments (*n* = 7), physical activity for chronic disease prevention (*n* = 6), health-related behavioral theory (*n* = 3), and urban planning (n =2).

Fifteen reported their level of agreement with the conceptual model. None of them reported complete disagreement, while two thirds (*n* = 10) reported agreeing with the evaluated version (agree = 8, completely agree = 2).

All comments regarding the conceptual model are available in the Additional file 2 (Table S2.2). The most common comments were related to: (1) replacing the construct “habit” with “behavior”; (2) the apparent much deeper coverage and complexity of the psychological attributes in comparison to environmental aspects in this version of the model; and (3) the need to deal better with the bi-directional relationship between intention and perceived environment for practice. Some experts noted that attitude and self-efficacy probably share most of the determinants considered in the model (e.g., perceived environment to practice would influence self-efficacy and attitude). Reviewers also noted the limitations in including socioeconomic status as the main influence on the built environment features.

In general, there were few suggestions to exclude elements or relations. Among the suggestions for inclusion, there were more proposals for new relations than new elements. This means that, overall, the conceptual model was judged to already include an appropriate and relevant set of constructs for the problem under study.

**Fig. 2:**
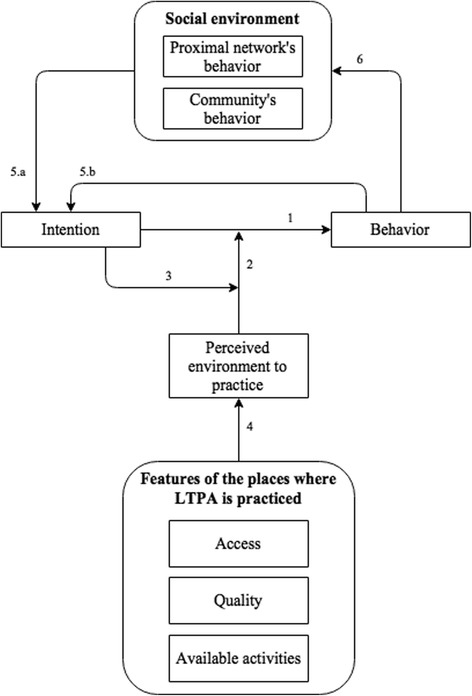
Conceptual model

**Fig. 3:**
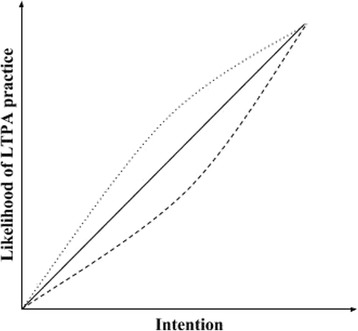
Example of the moderating effect of the perceived environment to practice on the relation between intention and the likelihood to practice leisure-time physical activity (LTPA). The continuous line refers to a relation with no effect modification. The dotted and dashed lines display how the relation between intention and likelihood of practice can be moderated by more positive (dotted) or negative (dashed) perceptions of the environment

The resulting conceptual model can be seen in Fig. 2. Tables 1 and 2 contain the operational definitions of the constructs and details about their relations, respectively. Figure 3 depicts the relationship between intention and likelihood of LTPA practice conditional to perceived environment (relations 2 and 3 in Fig. 2 and Table 2). Additional file 1 (Table S1.1) details all changes made between the first and the last version of the conceptual model.

**Table 1:** Operational definition of the constructs contained in the conceptual model

Domain	Construct	Operational definition	Reference
Individual attributes	Behavior	Individual’s LTPA practice during a certain period of time	-
	Intention	Effort the individual would employ to engage in LTPA	Hagger et al. [24]
	Perceived environment to practice	The individual’s perception of the existence of places where LTPA is practiced and their features	Nasar [31]
Social environment	Proximal network’s behavior	LTPA practice of the people within the person’s proximal network (friends, relatives etc.)	Carron et al. [32]
	Community’s behavior	LTPA practice of people living in a relatively large and geographically limited area	
Features of the places where LTPA is practiced	Access	How easily people can reach the place, including factors such as traffic, safety, physical proximity, cost and ease of transportation to it	Aytur et al. [33]
	Quality	Attributes such as maintenance, conveniences offered, aesthetics, equipment, lighting, security and layout	
	Available activities	Amount and types of activities available	

*LTPA* leisure-time physical activity

**Table 2:** Meanings and assumptions of the relations contained in the conceptual model

Relation	Meanings and assumptions
1	A person’s behavior is a function of his intention. The higher the intention, the more likely a person is to execute the behavior
2	The relation between intention and behavior is moderated by the person’s perception of the environment to practice. The more positive the perception, the more positive the relation between intention and behavior (Fig. 3)
3	The influence of the perceived environment to practice (relation 2) on the relation between intention and behavior (relation 1) has a U shape. The closer intention is to its upper or lower limit, the weaker the moderation effect of the perceived environment on the relation between intention and behavior (Fig. 3)
4	The person’s perception of the environment to practice is a function of the features of the places where LTPA is practiced. The better the features, the higher the likelihood of a positive perception
5 (a & b)	The person’s intention is a function of his previous behavior and the behavior of his proximal network and community. This influence has an inverted U shape. The closer intention is to its upper or lower limit, the weaker the influence of the person’s previous behavior and of the social environment on his current intention
6	The behavior of the proximal network and community are influenced by the person’s behavior

## Discussion

Our goal was to develop a conceptual model to inform and support the construction of an ABM aimed to understand the formation and evolution of population patterns of LTPA among adults. Our work will start focusing on the dynamic interaction between key psychological attributes of individuals and the built and social environments in which they live. The current conceptual model explicitly presents intention as the proximal determinant of LTPA, a relationship dynamically moderated by the built environment – with the strength of the moderation varying as a function of the person’s intention – and influenced both by the social environment and previous LTPA practice.

Another major contribution of this work is the process we used to develop the model. Researchers can follow or adapt the steps to develop their conceptual models for other behaviors. Moreover, it is worth highlighting that the conceptual model may be used by other researches for different purposes, such as informing their data collection and analysis and testing the correctness of the assumed relationships. Researchers may also expand, modify or adapt the model to their needs.

The conceptual model we developed has three important features. First, it posits dynamic mechanisms linking psychological attributes, aspects of the social and built environments, and physical activity. Generally, models and theories on the adoption and maintenance of physical activity include some, but not all of these aspects. When they encompass all these factors, as in the case of social-ecological frameworks, causal pathways between the elements are infrequently presented [4]. Second, our conceptual model is strongly based on the premises of the systems approach [27], adopting feedback loops and a dynamic perspective on the phenomenon, and assuming that one’s behavior influences and it is influenced by its perceived environment and other people actions, allowing responses and adaptations among heterogeneous individuals, which are, at the same time, autonomous and interdependent, generating complex system-level patterns and behaviors. Third, the model integrates current knowledge from published theories and models, empirical data, and expert opinion.

On the other hand, it is possible that not all relevant mechanisms and elements have been incorporated into the current conceptual model. This could have happened for three reasons. First, data may still too scarce or inconsistent on some aspects. Second, preference was given to certain mechanisms, especially those that generated feedback cycles, as our ABM must be based on the premises of the systems approach. Third, we purposefully started by a parsimonious conceptual model and ABM, even though many factors have been studied with varied levels of evidence supporting their association with LTPA. This is a recommended strategy in order to understand mechanisms and relations within the system’s dynamics [28]. Therefore, some information obtained from the literature review and experts’ suggestions were not included in the model’s current version, despite being relevant, but were instead archived for future use as the model evolves.

Our effort will extend beyond the current model. There still are space for improvement through the incorporation or elimination of elements and mechanisms. Based on the literature review and experts’ opinions, some suggestions already stand out as potential candidates for future improvements:

- The deconstruction of intention into its psychological precedents: attitude and self-efficacy. Both can also be broken down into their subtypes, such as task and barrier self-efficacy [29] or affective and instrumental attitude [30];
- Attributes of places where LTPA is practiced could also be deconstructed, for example, separating out financial cost of use and distance, within access;
- Incorporation of processes by which the social environment influences the attributes of places where LTPA is practiced, or the perception of these attributes at least;
- Influence of the behavior on the alignment between the objective and perceived built environment attributes;
- Inclusion of elements and mechanisms of the volitional phase, regarding the control over the behavior, such as goal definition and action plan [24, 30].

It is also important to note that the conceptual model is based on the best evidence available, but not all available evidence is equally consolidated, and some of the evidence available did not specifically refer to the leisure domain. Additionally, most of the evidence is concentrated in certain geographic areas (primarily high-income countries) with little data, for instance, from African and Asian countries. This partially reduces the reliability of the model’s assumptions and its capacity for generalization. While these points represent limitations of the current conceptual model, they highlight more general gaps in our knowledge about what influences population patterns of LTPA.

## Conclusion

The conceptual model we developed encompasses key elements involved in the formation and evolution of population patterns of LTPA among adults, emerging from the interaction between the psychological attributes of individuals and the built and social environments in which they live. Although simple, the current model is well supported by evidence and experts’ opinion and will enable us to grasp some of the mechanisms and relations within the system’s dynamics.

Our conceptual model will inform the design of our ABM, as well as data collection and analysis of future investigations on population patterns of LTPA among adults. Our next steps include developing and parameterizing the ABM using available data and validating it by contrasting outputs under various scenarios to patterns observed in the real world. Once validated, variants of the ABM might be used to test specific questions about plausible impacts of interventions.

## Additional files

Additional file 1: First conceptual model and changes made between versions. (PDF 177 kb)

Additional file 2: Version of the conceptual model assessed by experts. (PDF 416 kb)

Additional file 3: Results from the literature review. (PDF 166 kb)

## Abbreviations

LTPA: Leisure-time physical activity
