# Supplementary material for: Development of a dynamic framework to explain population patterns of leisure-time physical activity through agent-based modeling
Source: Int J Behav Nutr Phys Act. 2017 Aug 22;14:111. doi: 10.1186/s12966-017-0553-4 (PMC5568398; doi:10.1186/s12966-017-0553-4)
Supplement: Supplementary file 1 — First conceptual model and changes made between versions. (PDF 177 kb) [file 12966_2017_553_MOESM1_ESM.pdf]

Additional File 1. First conceptual model and changes made between versions.

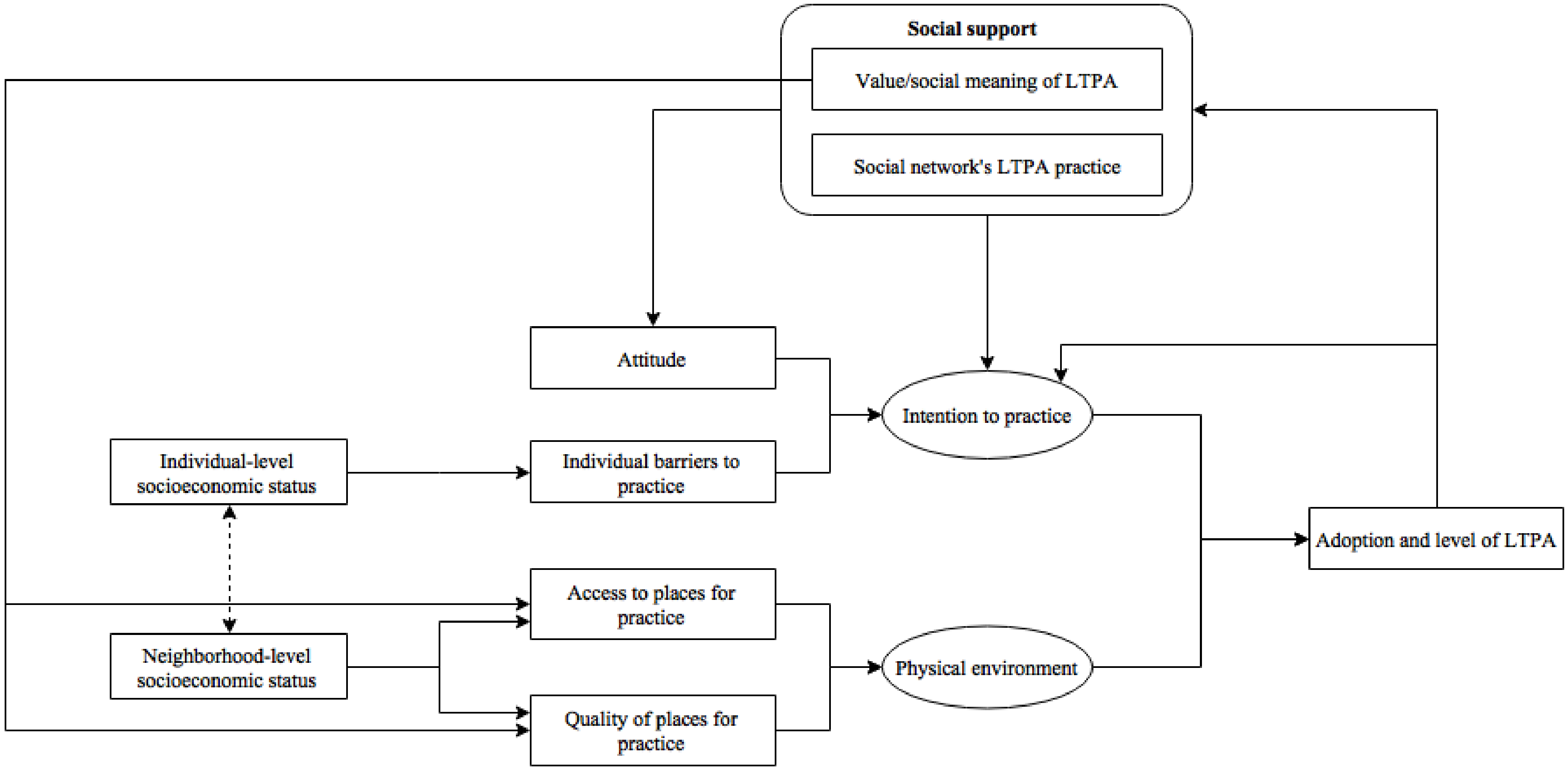

Figure S1.1. First conceptual model developed. LTPA = leisure-time physical activity.

Table S1.1. Changes made between versions of the conceptual model.

Versions	Changes
1 → 2	*Elements:*<br>- Individual-level socioeconomic status: removed<br>- Neighborhood-level socioeconomic status: removed<br>- Individual barriers to practice: changed to self-efficacy<br>- Intention to practice: changed to cognitive processes, encompassing the constructs attitude and self-efficacy<br>- Physical environment: changed to places for practice, encompassing the constructs condition (equivalent to quality in Version 1), access, and variability of opportunities<br>- Adoption and level of leisure-time physical activity (LTPA): changed to behavior<br>*Relations:*<br>- Behavior influencing intention to practice: changed to behavior influencing attitude (within cognitive processes)<br>- Social support influencing intention to practice: removed<br>- Social network's LTPA practice influencing self-efficacy: included<br>- Behavior influencing self-efficacy: included<br>- Value/social meaning of LTPA influencing access to places for practice: removed<br>- Value/social meaning of LTPA influencing quality of places for practice: removed<br>- Places for practice influencing behavior: changed to influencing the relationship between cognitive processes and behavior (moderating effect)
2 → 3	*Elements:*<br>- Socioeconomic status: included<br>- Cognitive processes: changed to intention. The constructs attitude and self-efficacy remained in the model, preceding intention<br>- Behavior: changed to behavior/habituation<br>*Relations:*<br>- Attitude influencing intention: included<br>- Self-efficacy influencing intention: included<br>- Socioeconomic status influencing self-efficacy: included<br>- Socioeconomic status influencing places for practice: included<br>- Places for practice influencing self-efficacy: included
3 → 4	*Elements:*<br>- Value/social meaning of LTPA: changed to community's LTPA<br>*Relations:*<br>- Places for practice influencing the relationship between intention and behavior (moderating effect): changed to places for practice influencing behavior
4 → 5	*Elements:*<br>- Behavior/habitation: changed to habit
5 → 6	*Elements:*<br>- Social network's LTPA: changed to social network's habit<br>- Community's LTPA: changed to community's habit<br>- Places for practice: changed to attributes of the places where LTPA is practiced, encompassing the constructs quality and condition, access, and available activities

Table S1.1. Changes between versions of the conceptual model (*continuation*).

Versions	Changes
6 → 7	*Elements:*<br>- Perceived environment for practice: included<br>*Relations:*<br>- Attributes of places where LTPA is practiced influencing perceived environment for practice: included<br>- Attributes of places where LTPA is practiced influencing self-efficacy: changed to perceived environment for practice influencing self-efficacy<br>- Attributes of places where LTPA is practiced influencing habit: changed to perceived environment for practice influencing habit<br>- Intention influencing perceived environment for practice: included
7 → 8	*Elements:*<br>- Habit: changed to behavior<br>- Social network's habit: changed to social network's behavior<br>- Community's habit: changed to community's behavior<br>- Attitude: removed<br>- Self-efficacy: removed<br>- Socioeconomic status: removed<br>- Quality and condition: changed to quality<br>*Relations:*<br>- Social environment influencing intention: included<br>- Behavior influencing intention: included<br>- Behavior influencing perceived environment for practice: included<br>- Perceived environment for practice influencing intention: included
8 → 9	*Elements:*<br>- Social network's behavior: changed to proximal network's behavior<br>*Relationships*:<br>- Behavior influencing perceived environment for practice: excluded<br>- Perceived environment for practice influencing behavior: changed to influencing the relationship between intention and behavior (moderating effect)
9 → 10	Review of the operational definitions, relations, design aspects, and introductory text

Removed elements also had their relations removed. LTPA= leisure-time physical activity.
