# Supplementary material for: Development of a dynamic framework to explain population patterns of leisure-time physical activity through agent-based modeling
Source: Int J Behav Nutr Phys Act. 2017 Aug 22;14:111. doi: 10.1186/s12966-017-0553-4 (PMC5568398; doi:10.1186/s12966-017-0553-4)
Supplement: Supplementary file 2 — Version of the conceptual model assessed by experts. (PDF 416 kb) [file 12966_2017_553_MOESM2_ESM.pdf]

Additional File 2. Version of the conceptual model assessed by experts.

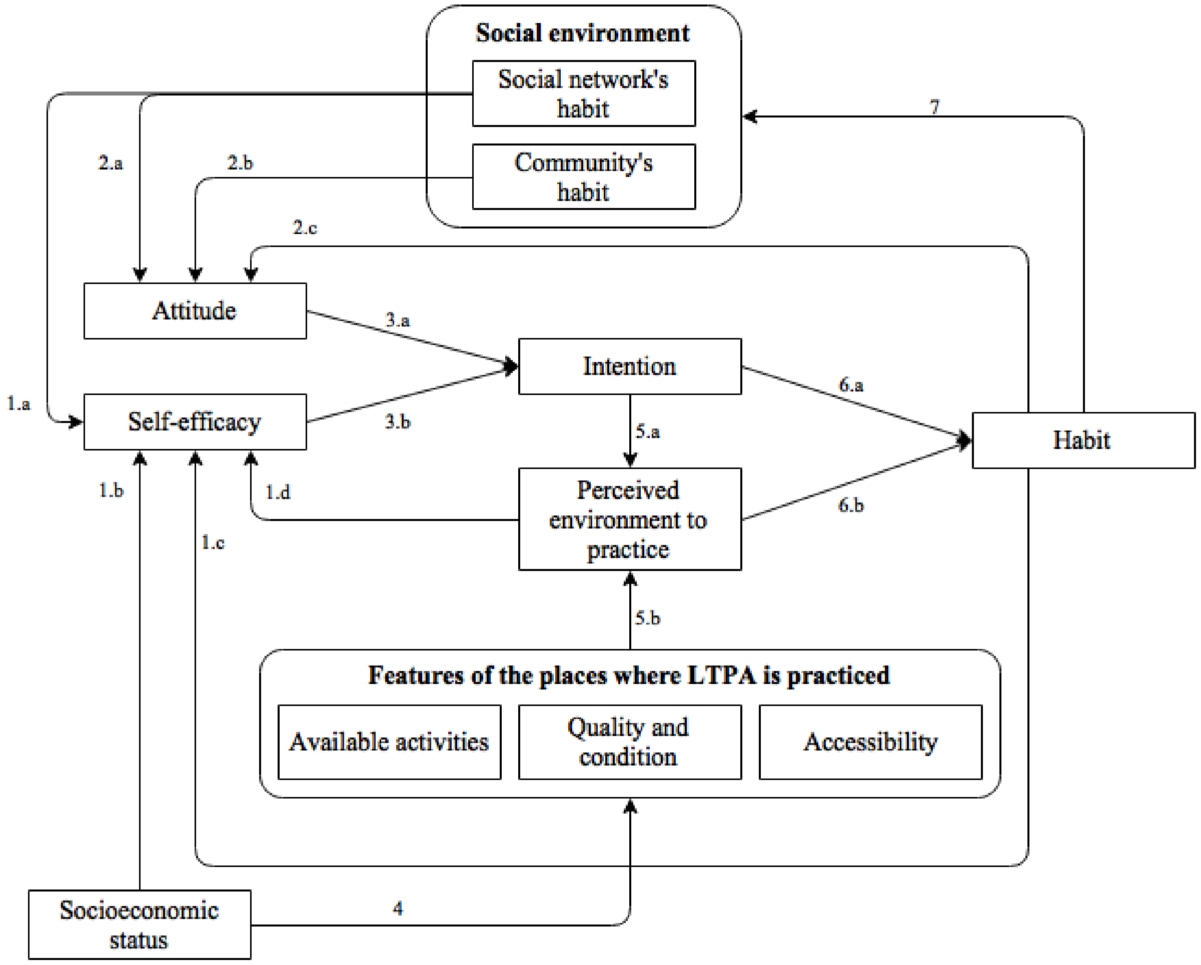

Figure S2.1. Version of the conceptual model assessed by experts. LTPA = leisure-time physical activity.

Table S2.1. Operational definition of constructs and meaning of the relationships contained in the version of the conceptual model assessed by experts.

**Model's delimitation:** our conceptual model tries to encompass the main psychological and environmental variables and mechanisms that might be involved in the emergence of collective patterns of LTPA practice among adults. Some aspects (such as demographic attributes and constructs related to the volitional phase of behavior adoption) were not included for the sake of simplicity, and because we feel they are not highly relevant to our research questions.	
1	*Self-efficacy* (person's self-perception about his/her competence to execute or control the behavior. It comprises barrier self-efficacy – confidence to overcome possible barriers to performing repeated bouts of physical activity – and task self-efficacy – confidence to perform the specific physical activity act itself) is a function of:<br>a) *Social network's habit* (leisure-time physical activity habit of those people with whom the person has proximal relationship; similar to social support), acting on barrier self-efficacy;<br>b) *Socioeconomic status* (person or group's economic and social position in relation to others), acting on barrier self-efficacy;<br>c) *Habit* (person's regularity of leisure-time physical activity practice), acting on task self-efficacy;<br>d) *Perceived environment to practice* (person's perceptions about available places for leisure-time physical activity practice and their features – available activities, quality and condition, and accessibility – combining spatial distance and affordability), acting on barrier self-efficacy.
2	*Attitude* (person's evaluation and expectation about the behavior and its outcomes. It comprises affective attitude – enjoyment and pleasure expected from physical activity –, instrumental attitude – utility of physical activity practice –, and social reaction – social approval or disapproval the behavior produces in one's interpersonal relationships) is a function of:<br>a) *Social network's habit*, acting on instrumental attitude and social reaction;<br>b) *Community's habit* (leisure-time physical activity habit of those people living in the same relatively large, geographically delimited settlement, such as a city; similar to social norm), acting on social reaction;<br>c) *Habit*, acting on affective and instrumental attitudes.
3	*Intention* (person's conscientious inclination to practice leisure-time physical activity) is a function of:<br>a) *Attitude*;<br>b) *Self-efficacy*.
4	*Features of the places where leisure-time physical activity is practiced* is a function of *socioeconomic status*.
5	*Perceived environment to practice* is a function of:<br>a) *Intention*;<br>b) *Features of the places where leisure-time physical activity is practiced*.
6	*Habit* is a function of:<br>a) *Intention*;<br>b) *Features of the places where leisure-time physical activity is practiced*.
7	*Social environment* (social network and community's habit) is a function of the *habit* of each person.

Table S2.2. Comments of experts on the conceptual model.

Expert	Agreement with the model	What to add in the model	What to remove from the model	Other comments
1	4	Important variables are included. I think the model is sufficient for initial analysis	No	Good model test it
2	4	More of an ecological framework, including the policy environment<br>Safety is another key issue to be included<br>Is this meant for Latin America or all countries/areas of the world? Would specify	Would change the term “habit” to “behavior”<br>I’m not certain what “perceived environment to practice” means<br>In general, the model covers psychological variables better than environmental/policy variables and the latter could be improved.	-
3	4	I’m not quite sure but time is sometimes a constraint to LTPA.<br>Secondly, it seems that acessibility may overlap the idea of facilities (having facilities), which, in fact are not the same.	-	Well done. Thank you
4	4	I think that ‘habit’ is developed after initiation of a behavior. In my opinion, intention is enough to initiate a behavior, but a setting that facilitates the behavior is needed for the maintenance/habit. Given that individual-level interventions focusing on motivation are often successful in the short term, but sustaining behavioural change is a real challenge, you may want to clarify that intention and environment play a complementary role in the process toward achieving a habit.	I am not sure about dividing ‘social environments’ into social network and community (these two sound similar). I wonder if you could simply list this as something like ‘social norm’.	Intention is considered to influence people’s environmental perceptions (5.a). I assume that people with and without intention see the environment differently. But, some particular settings may provide a cue for physical activity. In such a case, environmental perception may also influence intention.

Table S2.2. Comments of experts on the conceptual model *(continuation)*.

Expert	Agreement with the model	What to add in the model	What to remove from the model	Other comments
5	3	Overall this an interesting model and I appreciate the use of different theories and models as an attempt to explain potential interactions between psycho-social and built environment variables. However, I see a few limitations.<br>First, the assumption that socioeconomic chracteristics is the only explanation for built environment features is really simplistic. Policies at all levels (local, state and federal), community preferences, real state market and local geography are examples of variables that potentially affect the built environment and hence it might change peoples perceptions. Would you say if someone changes its socioeconomic status this will change perceptions of the environment? Or would it change the quality or the features within the surrounding environment? This is a major limitation in my perspective.<br>Secondly, perception of the environment is affected by previous experiences in the environment, which are also related to the time one has been living in that area or neighborhood. Therefore, these two variables should be better explained in the model.	I'm not convinced by the actual model and the references included that "habit" is a good description for the outcome variable. What do you want to predict? Participation in any activity? The time or the frequency of LTPA?<br>The definition of habit is not clear and I'm not even sure this is applicable to leisure physical activity since this is not a very stable behavior. For instance, if for any reason you feel ill and can't practice any PA for one or two weeks how would you classify this person? If you enjoy being active once a month doing a monthly field trip for camping would this be enough for you to be classified and having a PA habit?<br>This is critical and should be really refined or excluded to better reflect the outcome variable.	-
6	3	The interplay among various domains of physical activity should be considered. For example, someone who travels by foot for four miles each day (to and from work for example) may not be physically active in leisure time.	-	This is interesting work and should be continued. I do hope you also incorporate systems thinking into your model as it matures. Thank you for including me.

Table S2.2. Comments of experts on the conceptual model *(continuation)*.

Expert	Agreement with the model	What to add in the model	What to remove from the model	Other comments
7	2	My feeling is that you cover most of the relevant bases and that adding further constructs would probably result mainly in redundancy within the model.	I would tend to look at things somewhat more simply, in terms of opportunities provided by the physical environment and what other people observed are doing.<br><br>Variables that get close to what people would infer about their own behaviour, like self-efficacy and intentions, will always add a bit of exclamatory power to a model. It doesn't seem to me to be as interesting as trying to delineate the relevant environmental and social factors in more depth!!!!!!	I do find the model somewhat abstract, when it is describing a field in which there is a body of empirical evidence and some established frameworks.<br><br>It might be helpful to look specifically at what the evidence is regarding associations of particular environmental attributes with different physical activity behaviors.There is a nice conceptual paper by Sugiyama in MSSE and several reviews looking at evidence in this field
8	-	-	-	I have no suggestions for changes in the model. I think it's very successful, providing adequate dimensioning to fundamental aspects to behavioral change. It encompasses social network habits and community habits, but also perceived environment for practice.
9	4	-	In addition to the socioeconomic status, the model should include other demographic factors that are recognized as correlates or determinants of PA. Many of the connections pointed in the model are unidirectional, while in fact we might think about such linking in a bidirectional way.	I could not see any factor to be excluded.
10	2	I believe you are covering relevant variables and mechanisms. I only have small comments which I will address in the last space provided	You should not exclude any constructs	My only concern in the way you have developed your conceptual framework is related to the definitions of some of your constructs. For instance, the definition of social environment provided in the model, as a function of social network and community habit, I would add that it is also a function of socio-economic status.

Table S2.2. Comments of experts on the conceptual model *(continuation)*.

Expert	Agreement with the model	What to add in the model	What to remove from the model	Other comments
11	-	-	-	At a quick glance, here are some thoughts that initially come to mind in looking at your conceptual model:<br>• It appears, from one of the lines in the document in which it’s noted that you’re not specifically interested in the “volitional phase of behavior adoption”, this implies that you are interested in people that are already in some phase of being active? If so, then it would appear that you would be capturing how active or somewhat active people are shifting their choices of physical activity location/venue when new venues are added, as opposed to seeing how many begin some leisure physical activity?<br>• Proximity/convenience of getting to places for LTPA would be important, and it’s unclear whether this is currently captured in your model.<br>• In the general physical activity literature, self-efficacy has tended to be a better prospective overall predictor of physical activity as well as changes in PA relative to Planned Behavior Theory constructs (such as “attitudes” and “intentions”). Given that, I would suggest that you make sure to measure Self-efficacy well.<br>• In the social environment domain, can you capture media-based messages and signage, etc. in the community related to physical activity? (For instance, during the World Cup, there was a lot of media focused on athletics and physical activities which could have influenced, at least during that time, people’s overall LTPA).

Table S2.2. Comments of experts on the conceptual model *(continuation)*.

Expert	Agreement with the model	What to add in the model	What to remove from the model	Other comments
12	3	Missing other drivers of attitude including physical ability, dissonance between physical environment and physical abilities (different to self-efficacy)<br>Does social environment include social media, social norms, communication of social norms?<br>More specificity about attitude and how does this differ from intention?<br>Similarly, self-efficacy is perceived ability and control to do something - attitude would be a sub set of this?<br>More than perceived environment (you've dealt with this in the subjective steps earlier in the chain) by the environment it is actual influences, I believe<br>Drivers into intention to habit are relatively complex but from habit to social environment seems a bit limited	Sorry put everything in box one	-
13	4	I'm not sure about the pathway that one's habit affects the social environment, or at least I think this should be a reciprocal relationship.<br><br>Also, will your model be behavior-specific? LTPA is a very broad concept: walking for recreation, cycling for recreation, jogging, gym, sport... In my experience, the environment is not a great predictor of non-specific LTPA (see my early paper in SSM for the distance of decay parameters which shows that people will travel further to use LTPA facilities than those for walking).	No - I think you have covered the pathways well - could 5a also be bidirectional pathway (rather than unidirectional)? could there also be a direct pathway from features of the places where LTPA is practiced to self-efficacy and habit (rather than through perceived environment to practice?) – I'm not sure, but it could be direct and mediated through perceived - I guess it will depend on how you measure "perceived".	Nice survey - thank you and good luck

Table S2.2. Comments of experts on the conceptual model *(continuation)*.

Expert	Agreement with the model	What to add in the model	What to remove from the model	Other comments
14	4	Link between habit and perceived environment - Here I think there are possible bidirectional associations, in which the amount of PA you undertake may influence your perceived environment (i.e. you may have not been aware of activity opportunities in your neighbourhood, such as classes on offer, until you actively started looking for them to help maintain your PA).<br><br>Your introduction mentioned the interaction between env and psych variables, but the model does not reflect that (i.e. it is assuming a linear relationship between the variables). Recent work has started to show that PA-promoting environmental features may be more important for those with low attitude/intention, and so assuming that associations work the same for all may be a bit simplistic.	It may be useful to distinguish more between social network and community - they could overlap.	Good luck with the work.
15	4	I do not think new variables are needed. The problem is that the model needs to take into account accelerants, delays, competing actions, as suggested in some system approaches.	No.	I like the conceptual model, but think you should try to incorporate some system thinking into it.

Table S2.2. Comments of experts on the conceptual model *(continuation)*.

Expert	Agreement with the model	What to add in the model	What to remove from the model	Other comments
16	-	-	-	Leandro,<br><br>I have looked into your proposal, which is very daring, as appropriate for a Doctorate's.<br><br>I missed the influence of the transtheorical model (Prochaska). Does that not fit into your background?<br><br>Due to the influence of the stages of change, it seems to be a huge "leap" between intentions and environment perception and "habits". I believe that "trying out the practice" or doing so for an experimental period do not constitute a "habit" yet, but it's almost unavoidable in the process of behavioral change.
17 (continues in the next page)	5	1. Self-efficacy:<br>I consider that figure and the textual description are consistent.<br><br>2. Attitude:<br>The figure is clear and it has the necessary link. In the textual part, I believe that the item "Social network's habit" also acts on the "affective attitude", because social networks are formed mostly for the sake of collective practice. For example, some people like and feel more comfortable in performing collective PA due to the affective connection that originated this practice. I agree that the other degrees of attitude are contemplated here.	No, there aren't.	-

Table S2.2. Comments of experts on the conceptual model *(continuation)*.

Expert	Agreement with the model	What to add in the model	What to remove from the model	Other comments
17 (continuation)		3. Intention:<br>The arrows in the figure are well positioned. The only caveat I do is for ordination. I am not convinced that the attitude precedes the self-efficacy in the order of arrows! On the other hand, I think, attitude has a more critical input to generate an intention than just feeling able to execute or control a given behavior.<br><br>4. Features of the place where LTPA is practiced:<br>The figure and the textual description are consistent.<br><br>5. Perceived environment to practice:<br>I agree with the two mentioned paths and their order. I think the way one perceives the environment for the practice may be influenced by the social environment (i.e., by both social networks and the community where I belong). It may be worth testing the inclusion of an arrow from the social environment for the perceived environment to practice.<br><br>6. Habit:<br>I agree.<br><br>7. Social environment:<br>I agree.		

Table S2.2. Comments of experts on the conceptual model *(continuation)*.

Expert	Agreement with the model	What to add in the model	What to remove from the model	Other comments
18 (continues in the next page)	5	Thank you for the chance to review this conceptual model. I think it is a very interesting and important study you are doing and I am looking forward to the results.<br><br>Just some considerations came to mind. Please consider them as food for thought, not as concrete suggestions since I understand that some of these considerations may have already been discussed and decided upon during the development of the model:<br><br>1. Have you considered aesthetics of the environment? The characteristics that are now considered are more functional (availability, accessibility). Aesthetics is more personally determined which may make it more difficult to include in your model, but it may be important for physical activity in adults (see Bauman et al, 2012: doi:10.1016/S0140-6736(12)60735-1).<br><br>2. Have you considered to include perceived environment as a determinant of attitude? For example, when an environment is more attractive, being active in this environment may appear more pleasant, influencing affective attitude.	-	I was wondering about the influence of perceived environment on habit. Do you assume a direct relation or does the perceived environment act as a moderator between intention and habit? For example, when a person has a very positive intention but they perceive barriers in the perceived environment (e.g. no adequate facility nearby) they may not be able to turn this intention into action. On the other hand, if a person has a very positive intention and the facility is available, they are able to be active as planned. Is it possible to include interactions in your model?<br><br>Are the features of the places where LTPA is practiced considered to be constant (apart from the influence of SES but that is static since SES does not change in this model)? In other words, are people assumed to only be exposed to the PA places in their own neighbourhood/environment? Or are they allowed to go to other neighbourhoods/environments? For example, when people develop a PA habit, they may choose to move to a different neighbourhood with better facilities or they may travel further for better facilities.

Table S2.2. Comments of experts on the conceptual model *(continuation)*.

Expert	Agreement with the model	What to add in the model	What to remove from the model	Other comments
18 (continuation)		3. Would you consider habit as influencing the perceived environment as well? When people are more exposed to their PA-relevant environment (by being active in them), they may change their perception of this environment. Furthermore, it is possible that people who are not active judge the PA-environment less positive due to cognitive dissonance mechanisms? This last aspect may already be covered with the link between intention and perceived environment.<br><br>4. I noticed that subjective norm, one of the factors in the Theory of Planned Behaviour, is not included in the model. The concept of 'social reaction' within 'attitude' did seem to overlap with this concept. Did you merge these two concepts deliberately?		In the introduction of the model, it is stated that some constructs are not included in the model due to simplicity considerations. I understand that it is not possible to include everything but some of these factors do seem to be very important in determining physical activity and they may also interact with some of the other variables. Do you somehow adjust for the variation in these underlying determinants in your model? Especially health status (which is also socioeconomically determined) may be important to consider.<br><br>There is a small mismatch between the graphical representation and textual representation of the model: In the graphical representation, habit is a function of the perceived environment, wherein the textual format, it is said to be influences by the features of the places where ltpa is practiced. In my comments, I assumed the figure as correct.<br><br>I think it is a very well developed model and it contains most important features. I wish you good luck with your further research and I am looking forward to the results! Please do not hesitate to contact me if any of my comments is unclear or if you have additional questions.
