# Supplementary material for: Development of a dynamic framework to explain population patterns of leisure-time physical activity through agent-based modeling
Source: Int J Behav Nutr Phys Act. 2017 Aug 22;14:111. doi: 10.1186/s12966-017-0553-4 (PMC5568398; doi:10.1186/s12966-017-0553-4)
Supplement: Supplementary file 3 — Results from the literature review. (PDF 166 kb) [file 12966_2017_553_MOESM3_ESM.pdf]

Additional File 3. Results from the literature review.

Psychological aspects

Most psychological models and theories applied to physical activity define it as an intentional behavior, *i.e.*, that people deliberately engage in it [1], especially in the leisure domain. Intention may be interpreted as a construct that reflects the level of effort that someone would employ to perform a behavior [1-3].

Two meta-analyses have shown intention as the most proximal and strongest construct associated with the adoption and maintenance of leisure-time physical activity (LTPA) [4,5], having found effect sizes of 1.01 and 0.51, respectively. Other three meta-analyses [6-8], not limited to the leisure domain, have also found effect sizes varying from moderate to high, respectively 0.65, 0.51, and 0.43.

Models and theories predict that intention is preceded by attitude, perceived behavioral control (equivalent to perceived competence or self-efficacy [2]), and subjective norms. Attitude expresses the belief that the behavior will lead to certain desired results. Perceived behavioral control represents the belief and capacity to execute the behavior, as well as overcoming barriers using one's own resources. Subjective norms reflect the motivation to behave in conformity with what others expect [1-3].

Two meta-analyses suggest that attitude and perceived behavioral control are predictors of the level of intention to practice LTPA [4,5]. The effect sizes were, respectively, 1.07 and 0.60 for attitude, and 0.90 and 0.57 for perceived behavioral control. As for subjective norms, the effect sizes were smaller: 0.59 and 0.32, respectively. A third meta-analysis, not focused on LTPA, has shown similar results: 0.60 for attitude, 0.55 for perceived behavioral control, and 0.38 for subjective norms [8]. Additionally, during our review, past behavior stood out due to

its strong influence on intention, having similar effect sizes: 0.58 (only LTPA [5]) and 0.55 (several physical activity outcomes [8]), respectively.

Possible predictors of attitude, perceived behavioral control and subjective norms are less explored in literature, especially when referring to physical activity. However, theoretical assumptions [3] and a systematic review of studies carried out in the United Kingdom [9] indicated that the process of turning a casual behavior into habit influences attitude and perceived behavioral control. Further details regarding subjective norms can be found in the next topic.

Additionally, studies of Ding and collaborators [10] and Rech [11], both with LTPA, indicate that perceived behavioral control may also be influenced by one's perceived environment. Some authors, such as Lee and Cubbin [12] and Kamphuis and collaborators [13], also point out that socioeconomic status plays an important role on the levels of attitude and perceived behavioral control.

Finally, at least one meta-analysis [6], three systematic reviews [14-16] and one longitudinal study [17,18] indicated that among people with strong habit (*i.e.*, strong intention to maintain the behavior), the intention of keeping practicing physical activity seems to be less influenced by relapses, as well as other external factors, such as the physical and social environment. However, only one of these works (Rhodes and de Bruijn' systematic review [14]) specifically referred to LTPA.

Social environment

Several theories and models on the adoption and maintenance of health-related behaviors include constructs that represent the influence of the social environment on individual behavior [19]. The influence of the social environment may take place through norms regarding expected behaviors, social support (*e.g.*, enabling access to resources, information, and material for practice), having a companion during practice, encouragement and positive reinforcement from

others, and social learning and role modeling (*e.g.*, observing other people practicing and their means to overcome barriers) [9,20-22].

At least three meta-analyses [4,5,8] – the first two on LTPA – have shown that subjective norms seem to influence people's intention to practice physical activity, although to a smaller degree than attitude and perceived behavioral control (median value of effect sizes: 0.38 vs. 0.60 and 0.57, respectively). A systematic review by Weldel-Vos and collaborators [23] confirms these results, suggesting a positive association between LTPA and social support or companionship for practice in adults. Allender, Cowburn and Foster [9], in a systematic review of qualitative studies undertaken in the United Kingdom, have also concluded that the lack of social networks and realistic role models are barriers for participating in sports and general LTPA among adults.

Our review also revealed that the source of the social influence is important. Carron, Hausenblas and Mack [24] have observed, in their meta-analysis, that the effect size of social influence on the adoption of physical activity (usually LTPA) is, in general, greater when the source of the influence are people close to the individual (0.36-0.44 vs. 0.25-0.32).

A meta-analysis [6] and systematic review [16] have shown that social influence has a higher impact during the adoption than the maintenance stage. However, none of these works have assessed LTPA separately. On the other hand, at least three original studies that have used mediation analysis [25-27] have shown that attitude and perceived behavioral control mediated at least part of the effect related to the social influence on intention to practice LTPA.

Built environment

In order to present the results of this topic more easily, we organized them following the Aytur and collaborators' [28] conceptual framework on physical activity in outdoor community recreational environments. The first domain is access, representing how easily people can reach,

use and leave the place, including factors such as traffic, safety, geographical proximity, cost, and ease of transportation. The second domain is quality, reflecting features such as maintenance, conveniences offered, aesthetics, lighting, safety, and layout. The third is usability, referring to features that restrict or support people's engagement with the environment for a specific purpose, such as the amount and diversity of facilities and activities offered.

Access is the most investigated domain. However, only one meta-analysis could be found on the subject. Duncan, Spence and Mummery [29] have reported that adults who informed to have a place for physical activity practice in the neighborhood have shown 20% higher odds (IC95%: 6%-34%) of practicing physical activity, compared with who reported not having such a place in their surroundings. Despite not being restricted to the leisure domain, 73% of the studies included in this meta-analysis have investigated LTPA. At least four systematic reviews of quantitative [23,30-32] and one of qualitative [33] studies also reported a positive association between LTPA practice and objective or subjective measures of access, which involve the distance to or density of places where physical activity can be practiced. One of these reviews [23], however, suggested possible weak associations. Moreover, a systematic review focused on studies conducted in European countries [34] have not observed association between access, density or proximity to places where physical activity can be practiced and levels of LTPA.

As for quality, two systematic reviews have pointed out that the perception of conveniences offered, maintenance, overall condition, aesthetics, and safety were positively associated with LTPA [33,35]. On the other hand, Wendel-Vos and collaborators [23] have not observed association between physical activity practice and perceived aesthetics and safety, although observed positive association with conveniences offered.

Regarding the usability of recreational places, the amount and diversity of facilities and opportunities to practice physical activity have consistently shown positive associations to

LTPA practice in at least four systematic reviews [23,32,33,35], one of which dedicated to qualitative studies [33].

A review of reviews [36] found during our research has pointed out that social inequalities also seem to affect the access and quality of places for practice. In general, people living in more disadvantaged areas have reported more difficulties to access recreational places, as well as lower quality of these places. Moreover, access to these places seemed to influence the total physical activity level among these people in a much higher extent than it does among people living in richer areas.

A review conducted by Yang [37] has shown consistent evidence of an interaction between built environment and psychological attributes influencing LTPA practice. Based on the selected original studies, the author suggested that the built environment has a moderating effect, which influence is smaller on people with very positive or negative psychological attributes, resulting in a less powerful effect of the built environment on the likelihood to practice physical activity in such situations.

Finally, at least one original study [38] suggested that the perceived environment mediates the environment's influence on LTPA, and also that the subjective assessment of the built environment depends on its objective features. Another two original studies corroborate these findings, although they have investigated total [39] and transport-related [40] physical activity. On the other hand, one study did not observe this mediating effect of the perceived environment when investigating leisure walking [40].

References

1. Hagger MS, Chatzisarantis NL. An integrated behavior change model for physical activity. Exerc Sport Sci Rev. 2014; 42:62-9.

2. Michie S, Johnston M, Abraham C, Lawton R, Parker D, Walker A, et al. Making psychological theory useful for implementing evidence based practice: a consensus approach. Qual Saf Health Care. 2005; 14:26-33.

3. Montaño DE, Kasprzyk D. Theory of reasoned action, theory of planned behavior, and the integrated behavioral model. In: Glanz K, Rimer BK, Viswanath K, editors. Health behavior and health education: theory, research, and practice. 4th ed. San Francisco: Wiley; 2008. p. 67-96.

4. Downs DS, Hausenblas HA. The theories of reasoned action and planned behavior applied to exercise: a meta-analytic update. J Phys Act Health. 2005; 2:76-97.

5. Hagger MS, Chatzisarantis NLD, Biddle SJH. A meta-analytic review of the theories of reasoned action and planned behavior in physical activity: predictive validity and the contribution of additional variables. J Sport Exerc Psychol. 2002; 24:3-32.

6. Amireault S, Godin G, Vézina-Im LA. Determinants of physical activity maintenance: a systematic review and meta-analyses. Health Psychol Rev. 2013; 7:55-91.

7. McDermott MS, Sharma R, Andrews M, Akter S, Iverson D, Caputi P, et al. The moderating impact of temporal separation on the association between intention and physical activity: a meta-analysis. Psychol Health Med. 2016; 21:625-31.

8. McEachan RRC, Conner M, Taylor NJ, Lawton RJ. Prospective prediction of health-related behaviours with the Theory of Planned Behaviour: a meta-analysis. Health Psychol Rev. 2011; 5:97-144.

9. Allender S, Cowburn G, Foster C. Understanding participation in sport and physical activity among children and adults: a review of qualitative studies. Health Educ Res. 2006; 21:826-35.

10. Ding D, Sallis JF, Conway TL, Saelens BE, Frank LD, Cain KL, et al. Interactive effects of built environment and psychosocial attributes on physical activity: a test of ecological models. Ann Behav Med. 2012; 44:365-74.

11. Rech CR. A multidimensionalidade da atividade física de lazer em adultos: o papel dos aspectos intrapessoais, interpessoais e ambientais [Thesis]. Curitiba: Federal University of Parana; 2013.

12. Lee RE, Cubbin C. Striding toward social justice: the ecologic milieu of physical activity. Exerc Sport Sci Rev. 2009; 37:10-7.

13. Kamphuis CB, Van Lenthe FJ, Giskes K, Huisman M, Brug J, Mackenbach JP. Socioeconomic status, environmental and individual factors, and sports participation. Med Sci Sports Exerc. 2008; 40:71-81.

14. Rhodes RE, de Bruijn GJ. What predicts intention-behavior discordance? A review of the action control framework. Exerc Sport Sci Rev. 2013; 41:201-7.

15. Rhodes RE, Yao CA. Models accounting for intention-behavior discordance in the physical activity domain: a user's guide, content overview, and review of current evidence. Int J Behav Nutr Phys Act. 2015; 12:9.

16. van Stralen MM, De Vries H, Mudde AN, Bolman C, Lechner L. Determinants of initiation and maintenance of physical activity among older adults: a literature review. Health Psychol Rev. 2009; 3:147-207.

17. van Bree RJH, van Stralen MM, Bolman C, Mudde AN, de Vries H, Lechner L. Habit as moderator of the intention-physical activity relationship in older adults: a longitudinal study. Psychol Health. 2013; 28:514-32.

18. van Bree RJH, van Stralen MM, Mudde AN, Bolman C, de Vries H, Lechner L. Habit as mediator of the relationship between prior and later physical activity: a longitudinal study in older adults. Psychol Sport Exerc. 2015; 19:95-102.

19. Glanz K, Rimer BK, Viswanath K. Health behavior and health education: theory, research, and practice. 4th ed. San Francisco: Wiley; 2008.

20. Heaney CA, Israel BA. Social networks and social support. In: Glanz K, Rimer BK, Viswanath K, editors. Health behavior and health education: theory, research, and practice. 4th ed. San Francisco: Wiley; 2008. p. 189-210.

21. McAlister AL, Perry CL, Parcel GS. How individuals, environments, and health behavior interact. In: Glanz K, Rimer BK, Viswanath K, editors. Health behavior and health education: theory, research, and practice. 4th ed. San Francisco: Wiley; 2008. p. 169-88.

22. McNeill LH, Kreuter MW, Subramanian SV. Social environment and physical activity: a review of concepts and evidence. Soc Sci Med. 2006; 63:1011-22.

23. Wendel-Vos W, Droomers M, Kremers S, Brug J, van Lenthe F. Potential environmental determinants of physical activity in adults: a systematic review. Obes Rev. 2007; 8:425-40.

24. Carron AV, Hausenblas HA, Mack D. Social influence and exercise: a meta-analysis. J Sport Exerc Psychol. 1996; 18:1-16.

25. Boudreau F, Godin G. Participation in regular leisure-time physical activity among individuals with type 2 diabetes not meeting Canadian guidelines: the influence of intention, perceived behavioral control, and moral norm. Int J Behav Med. 2014; 21:918-26.

26. Grant F, Hogg MA, Crano WD. Yes, we can: physical activity and group identification among healthy adults. J Appl Soc Psychol. 2015; 45:383-90.

27. Zhang N, Campo S, Yang J, Janz KF, Snetselaar LG, Eckler P. Effects of social support about physical activity on social networking sites: applying the theory of planned behavior. Health Commun. 2015; 30:1277-85.

28. Aytur SA, Jones SA, Stransky M, Evenson KR. Measuring physical activity in outdoor community recreational environments: implications for research, policy, and practice. Curr Cardiovasc Risk Rep. 2015; 2015:423.

29. Duncan MJ, Spence JC, Mummery WK. Perceived environment and physical activity: a meta-analysis of selected environmental characteristics. Int J Behav Nutr Phys Act. 2005; 2:11.

30. Arango CM, Paez DC, Reis RS, Brownson RC, Parra DC. Association between the perceived environment and physical activity among adults in Latin America: a systematic review. Int J Behav Nutr Phys Act. 2013; 10:122.

31. Bauman AE, Reis RS, Sallis JF, Wells JC, Loos RJ, Martin BW, et al. Correlates of physical activity: why are some people physically active and others not? Lancet. 2012; 380:258-71.

32. Humpel N, Owen N, Leslie E. Environmental factors associated with adults' participation in physical activity: a review. Am J Prev Med. 2002; 22:188-99.

33. McCormack GR, Rock M, Toohey AM, Hignell D. Characteristics of urban parks associated with park use and physical activity: a review of qualitative research. Health Place. 2010; 16:712-26.

34. Van Holle V, Deforche B, Van Cauwenberg J, Goubert L, Maes L, Van de Weghe N, et al. Relationship between the physical environment and different domains of physical activity in European adults: a systematic review. BMC Public Health. 2012; 12:807.

35. Nasar JL. Creating places that promote physical activity: perceiving is believing. San Diego: Active Living Research; 2015.

36. Pearce JR, Maddison R. Do enhancements to the urban built environment improve physical activity levels among socially disadvantaged populations? Int J Equity Health. 2011; 10:28.

37. Yang Y. Interactions between psychological and environmental characteristics and their impacts on walking. J Transp Health. 2015; 2:195-8.

38. Van Dyck D, Veitch J, De Bourdeaudhuij I, Thornton L, Ball K. Environmental perceptions as mediators of the relationship between the objective built environment and walking among socio-economically disadvantaged women. Int J Behav Nutr Phys Act. 2013; 10:108.

39. Mama SK, Diamond PM, McCurdy SA, Evans AE, McNeill LH, Lee RE. Individual, social and environmental correlates of physical activity in overweight and obese African American and Hispanic women: A structural equation model analysis. Prev Med Rep. 2015; 2:57-64.

40. Jack E, McCormack GR. The associations between objectively-determined and self-reported urban form characteristics and neighborhood-based walking in adults. Int J Behav Nutr Phys Act. 2014; 11:71.
